# Factors That Influence, Exacerbate, Contribute or Promote Violence in Couples

**DOI:** 10.3390/healthcare11020281

**Published:** 2023-01-16

**Authors:** Claudia Sánchez, Cecilia Mota, Jorge Carreño, María Eugenia Gómez

**Affiliations:** National Institute of Perinatology (INPer), Mexico City 06600, Mexico

**Keywords:** intimate partner violence, symmetrical violence, asymmetrical violence, family abuse, affective deficiencies

## Abstract

Life as a couple, and their ability to solve problems or not, can result in the relationship being satisfactory and functional or unsatisfactory and dysfunctional, which increases the risk of violence with serious implications. For that reason, it is important to isolate the risk factors to prevent and treat unsatisfactory and dysfunctional relationships. The qualitative interpretive exploratory method was used, analysing 50 sessions of group psychotherapy with patients from a reproductive health institution whose relationships faced violent problems. The identified factors were the lack of autonomy in one of the partners, unresolved internal factors, a combination of external factors and factors caused by their interaction; symmetrical and asymmetrical violence, subjugation of one before the other, intergenerational violence, family violence during childhood and normalisation of violence. Isolating factors to understand relationship problems at risk of manifesting violence opens the possibility of effective, specific and preventive treatments of family and intimate partner violence.

## 1. Introduction

Life as a couple, and their ability to solve problems or not, can be random and unpredictable, and this can turn into a satisfactory and functional relationship or an unsatisfactory and dysfunctional one with the risk of triggering violence, thus compromising the physical and mental integrity of the couple. The Pan American Health Organization (PAHO, 2002) defines violence as:

“The intentional use of physical force or power, threatened or actual, against oneself, another person, or against a group or community, that either results in or has a high likelihood of resulting in injury, death, psychological harm, maldevelopment, or deprivation.

When restricted to interpersonal relationships, violence is divided into two: family or intimate partner violence, which occurs between family members or romantic partners, mainly at home with the intention of hurting or harming someone; and community violence, which occurs between unrelated people who may or may not know each other, and usually takes place outside the home” (p. 5–7).

In the presence of violence, there are also differences in intensity, which is shaped by culture [1,2]. That is why it is important to study it to establish an effective psychological approach, given that violence is the result of the couple’s interaction in their life together [3], because what should be a space for growth sustained with support and affection is at risk of becoming violent when conflicts are not resolved, leading to the control of one over the other, humiliations and blows that leave serious marks [4]. In a gender-perspective study conducted in Jalisco, Mexico, on women who were victims of violence [5], a higher incidence of psychological violence was found, followed by economic and physical violence. In another study conducted on women who presented psychological abuse compared to those who presented physical abuse to see the psychopathological repercussions such as anxiety, depression, post-traumatic stress and maladjustment in everyday life, no differences between the two groups were found in terms of high levels of these conditions [6]. These studies often sustain that violence is exerted by the man towards the woman; nevertheless, violence can also be exerted by the woman towards the man [7]. Some studies reveal that both women and men can equally suffer intimate partner violence, with higher scores of physical and sexual violence exerted by men and psychological violence exerted by both. In these studies, 34.1% of women and 26% of men fear their partner, for much before a knife or any other weapon is used, harsh words and tones are exchanged [8,9]. Moreno [10] found that spousal abuse occurs in both sexes, usually paired with a history of family violence during childhood, a process that is called intergenerational transmission where children replicate the violent behaviours or the passive role of their parents by normalising violent behaviours in their relationships [1]. Gelles [11] also identified the “cycle of violence” in couples, which is associated with child abuse. In Spain, young couples were studied and it was identified that verbal violence is the most frequent type of violence, followed by physical violence exerted by both sexes [12].

There is violence established as a form of permanent interaction that can last for years [13]. In a systematic review where the reasons for tolerating intimate partner violence were studied, it was found that men do not report it because this calls their masculinity into question and women do not report it for fear of the shame they could suffer when divorcing [14]. The study of intimate partner violence must avoid ideological biases [15] and have an integrative perspective [16], which cannot be accomplished through a partial approach, that is to say, that which only contemplates violence exerted by men and overlooks the psychological factors—fear, the need for affective bonds, emotional deficiencies during childhood—that determine violent behaviours which are neither masculine nor feminine [13,17,18,19,20]. In a study carried out to detect types and the frequency of violence in young people, the following types of violence were measured: emotional, physical, psychological, economic and sexual abuse. In the results, psychological abuse predominated, followed by physical abuse in adolescents and young adults [21]. There is evidence that shows that both women and men perceive violence from their partner. Moral et al. [22] point out that when conflicts are faced inadequately, they become chronic, which leads to fights, estrangement, heartbreak and finally violence, and that this is inherent to both women and men [23]. 

Having defined profiles avoids generalisations and stigmas, since there are studies where men report a higher frequency of violence received from women, which refutes the model of female victimisation and the male perpetrator [24,25]. On the other hand, Momeñe et al. [26], identified a positive correlation between emotional dependency and regulation and psychological abuse, concluding that the more that psychological abuse increases, the greater the emotional dependency on the partner. Avoidance coping in the face of violence has also been linked to an active role in the aggressor and a passive role in the victim, with no distinction between men or women [17,22,27]. Studies report that the complaints of male victims of domestic violence perpetrated by their female spouses have increased; however, the research related to this phenomenon is scarce. In a study where the presence of domestic violence in married men and those in a dating relationship was compared, it was found that married men suffer more psychological, social and sexual violence [28].

Haz [29] refers that spousal violence is any situation of abuse between the members of the couple in a cyclical way and with increasing intensity. It is a pattern of interaction that injures the physical, emotional and sexual integrity of the couple, and it is classified into (a) mistreatment against women; (b) mistreatment against men; and (c) reciprocal violence, for considering men as the perpetrators of violence against women because they have power and strength over them, who are vulnerable and fragile, is to consider women as incapable of achieving their autonomy and as perpetual minors [7].

At the National Institute of Perinatology (INPer) of Mexico City, an institution dedicated to the care of high-risk reproductive problems, an important service is that of psychological care, which receives patients who present—among other issues—relationship problems at risk of becoming violent while also dealing with a reproductive issue, but did not get any high scores in the violence detection protocols that may deserve some specific attention. Psychological intervention is required as part of comprehensive care because it is important to understand the interaction of the couple that can initiate the escalation of violence, which may sometimes become uncontrollable for the couple itself and whose results can be inconceivable and invade the family. Identifying the combination of factors that intervene in this destructive dynamic will allow the generation of intervention models that are preventive, decisive, and effective and focused on solving this problem that affects both members of the couple and their family, and that has great social impact. The treatment of choice is group psychotherapy for participants who refer to violence in the relationship with their partner, an effective tool whose fundamental purpose is to understand, through the analysis of group sessions, the factors that lead people to this destructive process in their relationship with a partner, in order to generate intervention strategies with greater specificity. For this reason, the following objective is proposed: to identify risk factors that influence, exacerbate, contribute or promote the violence detected during participation in a group psychotherapy process, where participants were included for referring to the experience of violence in their relationship.

## 2. Materials and Methods

The qualitative interpretative, exploratory method [30] was used, where the fundamental task of the researcher is to understand the complex world of evidential experience from the point of view of those who experience it and understand its various social constructions on the meaning of facts in an interactive process [31] with a narrative design [30], where the researcher [32] will collect data on the experiences of the participants to be able to describe and analyse them [33], against the stories presented in each of the sessions of group psychotherapy for couples with relationship issues at risk of becoming violent, which were the units of analysis that allowed the identification of the various factors that intervene in this phenomenon [34]. The research was carried out with the guidelines of the COREQ Reporting [35].

### 2.1. Scenario

The scenario was the Gesell dome of the psychology department at the INPer, where the group psychotherapy sessions for couples with relationship problems were captured, over a period of 18 months, with the following frame: two-hour sessions once a week with a fluctuating number of participants (between 10 and 14). The day, time and presence of the psychotherapist and their assistant were immovable and strict, and no interruptions were allowed. 

### 2.2. Sample

A convenience sample was used, whereby the capture guidelines were the previously established time—18 months—which in turn is the average time of stay at the institution for the participants. During this period, the registration of each session of the group process was carried out once a week; 50 sessions were captured during 2018 and 2019.

### 2.3. Participants

Participants are accepted into the institution due to a high-risk reproductive problem and are referred to the psychology department at the request of the patient, or because psychological problems with predominant relationship issues at risk of becoming violent have been detected during the admission process by the department of social work. Those patients who have psychological problems derived from their reproductive problems are referred to another type of psychological intervention. The women and men (the partners of the patients) who met the inclusion criteria were registered immediately after being referred to the psychological service, having already read and signed the informed consent letter. It must be noted that this group is permanently active so the patients receive attention and care independent of the time that the data recollection occurred for research purposes during the sessions. 

### 2.4. Inclusion Criteria

Adult patients newly admitted to the INPer who met the following criteria:

1. They faced violent problems in the relationship with their partner regardless of the reproductive health reasons for which they entered the institution.
2. All the women and men who were willing to participate in the group with relationship problems were included, and although the woman was the one who has been accepted for reproductive problems, the man was incorporated for studies when necessary. In the case of psychological service, they were invited to come to detect relationship problems where there might be violence.
3. They did not present a psychiatric problem for these patients were referred to specific assessments and management.
4. Those patients who did not want to take part in this research were referred to another group for receiving their required clinical care.

### 2.5. Procedure

A psychological interview was conducted on each participant.

Those who were candidates to enter the group received the informed consent letter from the psychotherapist, according to the institutional ethical requirements, to record in writing each group session. Subsequently, they joined the psychotherapy group specialised in relationship problems at risk of becoming violent, which is mixed, during the 18 months of data collection. All the subjects who decided to participate were included in the group process and it was explained to all of them that their treatment would be continued independent of the end of the study if they so required it.

To avoid bias, the observer—the one in charge of collecting the data in each session—and the psychotherapist—the one in charge of the group process—who attended all 50 sessions were always the same.

### 2.6. Ethical Considerations

The project was approved by the research and institutional ethics committees with the following registration number: 212250-3110-10810-02-16. The participants signed the letter of informed consent—approved by the Ethics Committee—where the steps to follow were explained to them, specifying that their data are anonymous, confidential and will be protected by the researcher for five years, as required by institutional rules, to be further destroyed in accordance to the anonymity of the participants.

This group is permanently active and independent of the sessions recorded for the present research; all subjects may continue the process until they are discharged.

### 2.7. Group Psychotherapy

The tool used to achieve the objective of this study was group psychotherapy, where group framing and technique are combined as it is an efficient and practical institutional work model [36]. A mixed group was formed where all participants referred to relationship issues at risk of becoming violent as their main problem and agreed to participate in the research. The group work allowed to focus on the processes and not on the isolated behaviours in order to identify the complexity that represents the interaction of the couple and the factors that contribute to the conflict between them and how the unsatisfactory elements in each one and their past experiences impact their current life together and cause dysfunctionality, thus leading to violence exerted by one or both members of the couple. The technique used was the one proposed by Bion [37], which conceives the group as a whole, where work is carried out on individual aspects while analysing each session globally within the group process [38]. All participation in the group work sessions is free and the psychotherapist’s interventions are based on the material gathered and shared in each session by means of the following: interventions that assess whether something happens in reality or is interpreted from reality; interventions to structure the couple; and interventions to work on their life history and current dynamics [39,40]. 

### 2.8. Data Collection Techniques

Fifty written sessions were analysed and consecutively listed and transcribed to facilitate their reading and subsequent analysis; the accounts of women and men were delimited. The sessions were recorded verbatim to make the first reading, which allowed for the systematisation of the information to subsequently make a second reading to describe and analyse it while respecting the narrative approach, as Creswell [32] reports, for telling a story helps to process issues that were not clear while looking for risk indicators in each session. 

### 2.9. Data Analysis

Central tendency measures were used to describe the participants. The measurements of women and men worked independently for the partners did not always agree to participate in the study. The material registered in each group psychotherapy session was analysed in terms of content with an empirical and exploratory orientation to categorise the most important elements based on three moments [41] with increasing progress: (a) a discovery phase, which consisted of repeatedly reading the data obtained from each session, to identify, locate and classify the risk indicators framed in the central objective, with an open system of categories developing a first field text; (b) a data coding and separation phase where the most repeated indicators were categorised by saturation for this is the moment where the information obtained begins to be the same, repetitive or similar [42], which leads to identify those of greater risk from where a second text with the manifested contents emerges, extracting the latent contents of each participant with the fragments of the most significant accounts based on four aspects: the theoretical ones detected in the systematised investigations of the couple’s problem with the presence of violence [43]; those observed in institutional clinical practice; the iteration of the participants’ indicators; and finally the minimisation phase, which is the interpretation and analysis of the data for it is where the contemplated categories and subcategories emerged. The final analysis considered the essential connections to isolate risk factors by grouping them. 

## 3. Results

### 3.1. Description of Participants 

The average age of the participants was 34 years for women and 35 years for men. The average schooling, measured in years, was 11 years for women and 12 years for men. The average group attendance during the 50 sessions that were analysed was 10.8 patients per session, with 60% of women and 40% of men attending, and an average of 25 sessions.

In relation to the reason for admission to the INPer for women, only that of women who are accepted into the institution, and the education of women and men, is captured (Table 1).

### 3.2. Data Retrieval

Four groups of risk factors for the escalation of violence were identified:

(1) Lack of autonomy as a family system.
(2) Problems with the inner world that were projected on the other.
(3) External reasons.
(4) Intimate partner violence due to interaction.

### 3.3. Risk of Violence Due to Lack of Autonomy

Three factors were identified:

First: The two members of the couple are dependent on their families of origin; they merge together with neither a life of their own nor a clear definition of their interaction, which affects their intimate life, which in turn is formed by their sexual life, communication and intimate space, which was either never built or is precarious. They have not managed to establish themselves as an autonomous system and they are embedded in an already constituted family system while absorbing their problems. The elements that hinder the process of independence are fear of failure, affective and economic dependence on the family of origin, and inability to make decisions regarding their life, their partner and their children. There is an association between abandonment, family abuse and affective deficiencies during childhood, which makes them feel vulnerable now that they are adults while being afraid of becoming independent and making decisions, which in the end creates conflicts derived from blaming each other and not acknowledging their part in the situation. Sometimes, they may find solace in one of the families of origin as an attempt to solve their problems, but without contemplating the possibility of emancipation, which is not an option. This in turn aggravates conflicts, intensifies and alternates complaints from one to the other, and leads to fear, anger and impotence, thus creating the perfect setting for manifestations of violence.

Second: One wants autonomy but the other one does not, and they acquire power over them for they have the support of their family of origin and do not want to lose it. Furthermore, the family has become their emotional priority and the main receptor of their time and money, and any negative action committed by them is minimised while being severely criticised when committed by the partner; this creates resentment and deteriorates the relationship as the partner is left in second place. This in turn provokes constant complaining because the decisions of their life together must be approved and consented by the family of origin with whom they are embedded and the member struggling for independence feels powerless, used, neglected and prone to be mistreated for they are seen as the enemy. 

Third: One struggles for autonomy but the other one cannot achieve it for they are subjected to their family of origin; they are submissive, which accentuates the risk of family abuse for the other perceives them as weak, insecure and unable to define their stand and avoid abuse. The factors that prevent their autonomy are (a) inability to face their family of origin for fear of rejection, estrangement, anger and breakup; they fear to be seen as traitors for not following the rules and breaking family complicity, which leads them to justify themselves before their partner and express their fear of not being able to obtain enough economic resources on their own without family support, thus feeling at a disadvantage when compared to others; (b) those related to childhood where they experienced abuse, abandonment and family rejection; they perceived themselves as an embarrassment to their parents and were constantly depreciated, with no defined position within the family; when becoming adults, this manifests as a difficulty of expressing desires, thoughts, needs, inconformity or anger, while always acquiescing to the desires of others; (c) they relinquish the control of their personal and emotional life, as well as their self-care, to the family of origin; they are constantly searching for maternal affection and struggling to have the place they never had in their family of origin, even at the expense of their independence, with the constantly repeated discourse of not having their own place or role in the world, which is something they felt from a very young age. They adapt and subject the family to their needs and incorporate the very same dynamic with their partner, with all the problems this entails, which in turn may intensify and result in manifestations of violence due to the lack of solutions. Footer of Table 2 are the stories of a woman and a man who exemplify the above. The others examples can be seen in Supplementary Materials (Table S1).

### 3.4. Risk of Violence due to Problems with the Inner World Projected on the Other

Problems were identified because the relationship is based on assumptions of what the other is, which leads to an overinterpretation of what they do. This in turn has more to do with inner conflicts and fragilities than with reality, and these are manifested at all times for the other is seen as an enemy. Therefore, the fractures in the couple are evident and they worsen when one of them faces painful experiences and inner issues such as the loss of a family member or problems at work. The way they face these incidents as a couple—for they impact both of them—will demonstrate the dysfunctionality of the couple and will worsen the conflicts due to the partner’s lack of expected answers, understanding and support, for they already have preconceived ideas of how the other should react or prove it, which contributes to assumptions on what the other feels. This in turn aggravates the crises and causes disappointment, anger and rejection due to feeling misunderstood, thus attacking the partner for not receiving the expected support from them. The attacks include cancellation of their sexual life, indifference, hatred, reprovals, isolation and emotional estrangement, all of which deteriorate the relationship, turning it into fertile ground for any manifestation of violence. Footer of Table 3 are the stories of a woman and a man who exemplify the above. The others examples can be seen in Supplementary Materials (Table S2).

### 3.5. Risk of Violence Due to External Reasons (Infidelity, Infertility and Perinatal Losses)

There are two factors that have a devastating effect on the couple:

First: The suspicion or certainty of infidelity, which creates a dilemma in the one who suspects or uncovers an infidelity, for every decision made in this situation is conflictive. The couple is left inevitably broken and it enters a process of instability and destruction that establishes itself as their way of life and is present at all times in the form of mistrust, constant rows, reprovals and endless arguments where everything else is secondary. The consequences are neglecting the children (if any), neglect of their health and quality of life; every aspect is invaded by this problem and the relationship suffers a gradual decline for everything is now tied to the infidelity. One begins to chase the other, which is unavoidable due to the constant uncertainty becoming more intense and frequent, thus underlying the violence. A serious element observed in these couples is that “the third party” becomes part of the relationship and their ghostly presence lives with them. All the discourse loaded with claims and complaints refers to this third person and the predominant symptomatology is jealousy, anxiety and a feeling of exclusion.

In the accounts of women, it was found that they had grounds for their suspicions of infidelity given that the partners themselves provide them with elements to suspect that there is another relationship. In men, the accounts show jealousy towards their partner, usually unfounded, which becomes part of the interaction. They look for details that can support their suspicions and they recognise that this jealousy may be caused by family inheritance, since their father and grandfather were the same and jealousy is part of their life story. 

Second: The by-products of reproductive health, which are frequent due to the type of patients/participants in the study include couples with infertility and couples with miscarriages or perinatal losses. Both situations have different nuances even if they’re psychologically impactful for the couple facing them.

- Infertility: The World Health Organization (WHO) [44], states that “infertility is a disease of the male or female reproductive system defined by the impossibility of achieving a pregnancy after 12 months of having unprotected sex” (14 September 2020). The couple faces several types of suffering: the first one is undergoing clinical studies to study the causes of infertility which is in itself an exhausting process; the second one is the suffering caused by family and social pressure, which aggravates their emotional state because there are situations that the couple goes through from which they cannot escape. These are of a high degree of violence because the couple is stigmatised and faces painful situations that constantly remind them of their inability to become parents, which will affect their intimate life and negatively impact their daily and sexual lives. If these couples had any previous conflicts, these may complicate further, as referred to by Mahmoodi [45] in his comparative study between fertile and infertile women, where it was found that infertile women have lower psychological well-being and suffer from more relationship problems.
- Miscarriages or perinatal Losses: The World Health Organization (WHO) [44] defines them as follows: Perinatal mortality is understood as the number of prenatal deaths and deaths during the first week of life (early neonatal mortality), but the definition of prenatal mortality varies and includes prenatal deaths after 22 or 28 full weeks of gestation (p. 21). Great suffering is detected for the loss of the baby and the expectations placed in both the pregnancy and the new being. This generates anger, pain and sadness, as they do not understand the reason for the loss and it leaves them feeling a great emptiness and guilt caused by the constant doubt of having done something wrong. However, there is less family and social stigma, as they receive a lot of support from their family and social networks. In both situations, the couple makes use of their adaptive resources, which are not always that successful because these situations end up invading various aspects of their lives in ways that can fracture the relationship or by projecting the other or themselves from their guilt, feeling devaluation and shame before others and overinterpreting the partner’s behaviour, which predisposes violence. Footer of Table 4 are the stories of a woman and a man who exemplify the above. The others examples can be seen in Supplementary Materials. (Table S3).

### 3.6. Risk Factors That Trigger Intimate Partner Violence Due to Interaction

Two types of manifestations of violence were identified with a gradual deterioration of the relationship and an increase in the risk for violent interaction. 

First: A predominance of symmetrical violence where both members mistreat each other with the intention of harming themselves through economic, psychological, physical violence or blackmail, which can lead to an escalation of violence too difficult to control, endangering their psychological and physical integrity. The predominating accounts are those where polarised and intense reactions in the relationship are identified, ranging from expressions of love to intense hatred. This can lead to physical violence because the transition between one type of violence and another is unpredictable and provoked by insignificant issues with no justified cause. This can have devastating consequences due to the difficulty of controlling it.

In both men and women, a lack of love during childhood and neglect predominate and they are both activated in adulthood in their relationship with the partner, where it finds a way to manifest itself. They recognise that their reactions towards the partner are exaggerated and disproportionate in relation to the stimulus that caused the violent outbreak.

Second: A predominance of asymmetrical violence where one submits to the other and the power is concentrated in one over the other. The latter has to obey with no claims and no power to make decisions, which places them in the background within the couple and family dynamics.

Recurrent discourses are identified where impotence and anger predominate for not being able to change their current circumstances. They feel unable to confront their spouse due to paralysing fear and a feeling of inferiority for the concept they have of themselves is mediated by what their partner tells them they are, always in terms of devaluation. It is because of that poor valuation of themselves that the dominant one consolidates his power. They turn into an object for the other, who does not tolerate them to have thoughts of their own and express their needs, discontent or dissatisfaction. They experience this as a threat and aggression, which increases violence because any manifestation of disagreement or “disobedience” from the subject unsettles the dominant, which in turn increases the paralysing fear and makes them feel helpless without knowing how to respond. There are similar elements between women and men in terms of their family dynamics of origin that condition their responses in adult life, such as the violence they experienced during childhood, abandonment, detachment, devaluation and affective deficiencies with the feeling of having no place in the world.

The consequences in their adult life are manifested in their choice of partner and in the tolerance to be mistreated or to mistreat their partner. Footer of Table 5 are the stories of a woman and a man who exemplify the above. The others examples can be seen in Supplementary Materials (Table S4).

**Figure 1:**
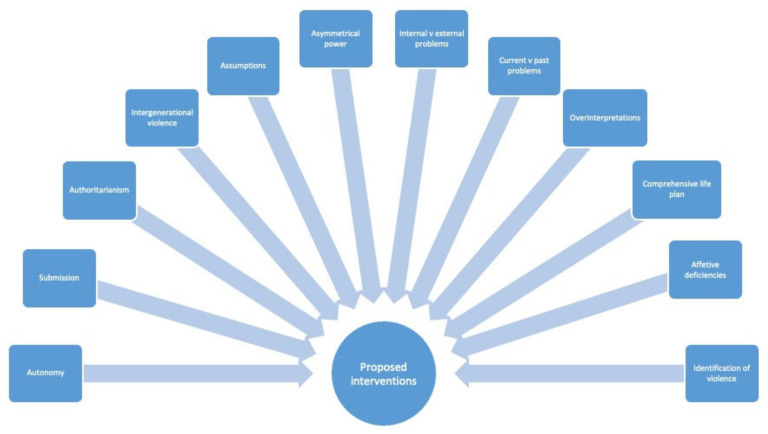
Proposed interventions.

The proposed interventions based on the factors that were identified in this study to have greater specificity in the interventions with this population, are presented in Figure 1.

## 4. Discussion

The objective was to identify risk factors that contribute to enriching intervention strategies with greater specificity for a serious problem that afflicts society: intimate partner violence. Due to the complexity and emotional burden of this relationship, when dysfunctional, it can become a weapon where members enter an escalation of destruction that must be detected and stopped on time by professionals—in this case, from the health sector—in order to offer timely attention and stop this process.

The manifestations of violence, as identified by González [1], have an element that distinguishes them from other couple conflicts: the clear and conscious intention to harm the other, which can be exerted by both women and men, in a symmetrical or asymmetrical way and can be a consequence of past experiences. They can be caused by external elements or by the interaction between them and sometimes this violence is unclear and is masked with different manifestations [8,9].

In terms of the risk factors and those that influence intimate partner violence, the first group coincides with what was brought up by González [1], Moreno [10] and Gelles [11], which is the violence experienced in childhood, such as abandonment, detachment, devaluation and affective deficiencies with the feeling of having no place in the world [46]. It is manifested as a repeated model in the relationship with one’s partner due to their inability to have a life of their own, to make decisions and to achieve their autonomy and growth. This leads them to depend on the family of origin and puts both the family bond on which they depend and the couple bond at risk, as reported by Ponce et al. [47], who related emotional dependency with marital dissatisfaction and the presence of violence, due to dysfunctional patterns of affective bonding along with a fear of loneliness, the need for affection and poor life satisfaction. This in turn increases unpleasant emotions with indifferent, hostile and violent attitudes that cause distress, fear, guilt and frustration, thus triggering depressive and anxious symptomatology [48,49,50].

The second group identified are internal and external factors that put the couple at risk. The internal factors are related to the preconceived idea of what the emotional or supportive demonstrations of one should be like when the other is going through a crisis that has to do with individual issues, which provokes an overinterpretation of the reactions of the other that have more to do with unresolved sources of conflict that lead them to place difficult demands on the other. Regarding the external factors, a serious conflict is the discovery or suspicion of infidelity, for the couple becomes trapped in a dynamic that invades all areas of their lives and any further decision made is conflictive and full of resentment due to the devastating impact of this circumstance. As for infertility, the characteristic is the mistreatment suffered by the environment for not complying with the social expectations of achieving a pregnancy. One of the nuclei that inflicts the most suffering is their family followed by their immediate social environment, which makes them feel helpless and turns them into victims of violence, since this is added to their already fragile psychological state caused by facing a gruelling process that tests the strength of the couple regarding the decisions they have to make, and this reproductive problem may increase marital dissatisfaction which has an impact on psychological health, which coincides with Edalat et al. [51], who notes that helping infertile women with their relationship problems improves their mental health.

In gestational or perinatal losses, guilt predominates, which translates into self-abuse and crossed mistreatment caused by entering into a painful crisis and the difficulties in developing grief, which can lead them to a depressive state by not finding an explanation for this painful event.

The third group identified coincides with what was brought up by Cienfuegos [13] and is the presence of partner violence as part of the daily interaction, not caused by an isolated fact but already established as part of their daily life. In this case, violence is normalised and can be symmetrical (the two are violent) or asymmetrical (one submits to the power of the other), which leads to question why a person can tolerate violence for years [19]. This coincides with Aiquipa [18], who refers to it as the conjugation of different factors, many of which are found in this study, that explains the tolerance to live in a relationship where violence predominates in different manifestations and which profoundly affects the lives of people with, at times, catastrophic consequences.

## 5. Conclusions

A fundamental element in the psychological care for these couples is that any ideological bias that may interfere with the objectivity of the treatment plans should be avoided, since labelling the man as the perpetrator and the woman as the victim hinders obtaining real help in the care provided to these couples. Furthermore, it denies that psychological factors are the ones that determine the interaction and presence of violence in couples; therefore, it is necessary to identify them and study their causes to generate a clear and precise approach [13,16,20,28].

The limitations of the study are that the studied population has specific characteristics and suffers from a high-risk health problem. Replicating studies in different populations would expand the knowledge about the risk factors that intervene in intimate partner violence. Another limitation is not taking into account couples who are at different stages of their life cycle to know if the behaviour in terms of violence is presented in the same way at different ages or circumstances. Isolating factors to understand these processes opens the possibility of effective, specific and preventive treatments of family and intimate partner violence.

## Figures and Tables

**Table 1:** Reproductive problem and schooling of the participants.

Reproductive Problem Women	%	Schooling in Grades	Women	Men
Risky pregnancy	15	Primary school	14%	4%
Infertility and perinatal mourning	40	Secondary school	30%	39%
Gynaecological problem	35	High school	29%	24%
Menopause	10	Bachelor’s degree	26%	31%
		Postgraduate studies	1%	2%

**Table 2:** Risk factors of violence due to lack of autonomy.

Categories	Subcategories	Elements Found
1: Both members of the couple depend on the family of origin	a. Repeated individual factors in childhood that impede growth b. Affective factors that prevent decision-making to achieve autonomy	Dependence on the nuclear family Abandonment and lack of affection during childhood Conflicts between the family of origin and the current family Dependence on other family systems Flaws in the family structuring
2: One wants autonomy, but the other does not	a. The critical emotional bond is with the family of origin b. The partner is trapped	The partner is in second place. Resistance to being independent Despair at the partner’s attitude The commitment is to the family, not to the partner
3: One struggles for autonomy, but the other one cannot achieve it	a. Submission to the family b. Causes of submission to the family	Limitations caused by dependency Anger and inability of the partner Child dependency of the partner Anger at the husband’s link to the family Indicators of child dependency

Woman: ‘I spend most of my time at my mum’s house; this generates conflicts with my partner. My guilt has intensified—my husband’s tongue is very sharp, he scares me’. Man: ‘The relationship with my partner and her family shows me the place everyone has. My wife demands too much of me but not of her family. She’s fine with whatever her relatives do but whenever I do it, she gets mad and scolds me. She doesn’t treat us the same’.

**Table 3:** Risk factors of violence due to problems with the inner world projected on the other.

Categories	Subcategories	Elements Found
1: Overinterpretation of the other’s behaviour	a. Paralysing fear b. Painful manifestations when facing a real event and disagreement with the partner c. Impotence for not knowing how to behave d. Acting on assumptions	Internal fears that prevent regulating the other Lack of communication and understanding from the other Despair at the other’s behaviour Impotence when facing a problem of the partner’s Attribution of health problems to couple problems The pain of a loss is transformed into anger The other does not meet expectations The children become the repositories of the problems Intergenerational alliances Childish attitudes in the relationship with the partner Overinterpretations
2: Relationship between the conflicts of their previous life and the conflicts with the partner	a. Internal voids b. Unresolved inner conflicts c. Inner dissatisfaction projected on the couple d. Problems projected on the children	Form of escape-avoidance coping Inability to defend oneself Impotence when facing the attitudes of the other Impotence when facing the wife’s behaviour Impotence when facing the other’s dissatisfaction Useless effort Lack of regulation

Woman: ‘I feel assaulted and misunderstood by my husband regarding my illness (depression); he doesn’t understand it, I feel it as an aggression’. Man: ‘I sense that my wife is dissatisfied with our relationship. Nothing I ever do seems to be okay for her, I never bring her any happiness’.

**Table 4:** Risk factors of violence due to external reasons.

Categories	Subcategories	Elements Found
1: Repeated behaviours following the other’s infidelity	a. Uncertainty	Inability to get out of the situation Passivity Inability to make decisions. Psychological violence
2: Identifying how they react to infidelity	a. Contradictions	Recurring ideas when facing a suspicion of infidelity Atrocities Anger deposited on the victim
3: Risk factors triggered by infertility problems	a. Losing control over the other generates anguish b. External problems that occur due to infertility	This promotes mistrust and control over the other Pain from infertility and abuse. The social surroundings worsen the pain Exhaustion caused by facing infertility The sexual life is annulled Envy before what is considered unfair
4: Facing perinatal losses	a. Devastation caused by the losses b. Impact on the couple relationship	Fragility when facing a painful event Stages of grief Pain caused by the loss The pain of a loss is transformed into anger The expression of pain as aggression

Woman: ‘What I felt is over. Now I don’t even want to see the light because I can’t have children. My mother-in-law believes I’m the one with the problem and all responsibility has been thrown at me’. Man: ‘I can’t seem to accept the pain for the loss of my baby. I blame my wife for not taking care of herself during the pregnancy, she’s diabetic’.

**Table 5:** Risk factors that trigger intimate partner violence due to interaction.

Categories	Subcategories	Elements Found
1: Violence within the family of origin influences intimate partner violence	a. Intergenerational violence b. Maternal rejection	Models of abuse during childhood repeated with the current family Normalised family violence during childhood Mistreated grandmother, mother and daughter Abusive mother due to dissatisfaction Consequences of the domestic violence Emotional deficiencies during childhood and resentment Rejecting the mother for being a woman; defencelessness The conflict with the mother is repeated with the daughter
2: Escalation of violence	a. Symmetrical violence b. Asymmetrical violence (one subjected to the other) c. Identifying elements that are triggered when on tries to be or becomes autonomous	Polarised relationships Causes of entrapment in an abusive relationship Impotence when facing abuse One imposing over the other Fear, insecurity, feelings of devaluation. Inability to modify their life Exclusion in collusion with the children Submission when facing intimate partner violence Domineering attitudes from an authoritarian woman Resentments poured on the partner Abuse when autonomy is achieved Authoritarianism and absolute control Harassment, control Control and isolation Increase in violence due to changes in the victim
3: Tolerating abuse	a. Repercussions in children b. Factors that prevent the separation of one member of the couple despite wishing for it	Passivity when facing the other’s aggressions towards the children Limitless efforts to please to other Inability to set boundaries Factors that prevent decision-making Submission as a high-risk characteristic in the couple
4: Abuse that goes beyond the couple relationship	a. Self-abuse and abuse towards the family b. Physical separation doesn’t always change the bond c. Normalising violence	Self-abuse Devaluation, self-abuse and self-destructive fantasies Lack of self-regulation The separation not always changes the couple dynamics Repetitive behaviours in spite of the separation Normalisation of violence Starting to uncover attitudes

Woman: ‘The problems I have with my family remind me of when my brother mistreated me. My mum didn’t say anything. I come from a small town and we were taught to respect the eldest sibling. I see my husband acts the same way my mother used to and my son is like my brother because he mistreats my daughter’. Man: ‘I give everything I have to my wife and children. She administers the money we both earn. Nevertheless, if I take some for an emergency, my wife complains, abuses me and treats me like her servant’. Examples of the participants’ stories; the others can be seen in Supplementary Materials: Table S1, Table S2, Table S3 and Table S4.

## Data Availability

Because this article was the product of an analysis derived from psychotherapy sessions, there is no database that shows their analysis. All the data obtained are the reports of the participants of the sessions that are described in the Supplementary Materials.

## Supplementary Materials

The following supporting information can be downloaded at: https://www.mdpi.com/article/10.3390/healthcare11020281/s1: Table S1. Risk factors of violence due to lack of autonomy; Table S2. Risk factors of violence due to problems with the inner world projected on the other; Table S3. Risk factors of violence due to external reasons; Table S4. Risk factors that trigger intimate partner violence due to interaction.
