# Supplementary material for: Factors That Influence, Exacerbate, Contribute or Promote Violence in Couples
Source: Healthcare (Basel). 2023 Jan 16;11(2):281. doi: 10.3390/healthcare11020281 (PMC9859608; doi:10.3390/healthcare11020281)
Supplement: Supplementary file 1 [file healthcare-11-00281-s001.zip › healthcare-1971688-supplementary.pdf]

healthcare MDPI

Supplementary Files

Table S1. *Risk factors of violence due to lack of autonomy*

Elements found	Units of analysis and accounts
**Category 1: Both members of the couple depend on the family of origin**	
**Subcategory a. Repeated individual factors in childhood that impede growth**	
Dependence on the nuclear family.	Woman. 'I spend most of my time at my mum's house; this generates conflicts with my partner. My guilt has intensified - my husband's tongue is very sharp, he scares me'.
Abandonment and lack of affection during childhood.	Woman. 'Conflicts with my parents affect me. My mother's abandonment created a conflict in me that I have not been able to resolve'.
**Subcategory b. Affective factors that prevent decision-making to achieve autonomy**	
Conflicts between the family of origin and the current family.	Man. 'My mother magnifies the facts in a negative and aggressive way. I understand my wife because of the problems she has had with my family. I find myself in a conflict between my wife and my mother'.
Dependence on other family systems.	Woman. 'I had a hard time to get pregnant and I was finally able to; nevertheless, my son is hospitalised due to health problems. I have problems at home because we don't have a space of our own. We don't work either; we economically depend on our relatives'.
Flaws in the family structuring.	Man. 'We've faced difficulties with our son's disease, it's creating us a lot of problems and so does me and my wife's unemployment. We're alternating houses every week: one week we stay at her parents' and the following week at mine's, and so on'.
**Category 2: One wants autonomy, but the other does not**	
**Subcategory a. The critical emotional bond is with the family of origin**	
The partner is in second place.	Man. 'I don't like fighting with my family. I'm just focused on fulfilling my chores. We work the land together. My wife is upset because of my close relationship with my family for we live with them'.
Resistance to being independent.	Man. 'The activities I do at work prevent me from moving house for we live with my parents. I have many things at their place'.
**Subcategory b. The partner is trapped**	
Despair at the partner's attitude.	Woman. 'It discourages me to see no attempts or results to become independent from his family. He doesn't seem interested and I pressure him a lot, I want my own space'.
The commitment is to the family, not to the partner.	Man. 'The relationship with my partner and her family shows me the place everyone has. My wife demands too much of me but not of her family. She's fine with whatever her relatives do but whenever I do it, she gets mad and scolds me. She doesn't treat us the same'.
**Category 3: One struggles for autonomy, but the other one cannot achieve it**	
**Subcategory a. Submission to the family**	
Limitations caused by dependency.	Woman. 'I resent the way my husband's family mistreat him and I'm angered by how my father-in-law acts towards him. My husband does everything they tell him, he's very passive. If I tell him about it, he evades me and gets upset'.
Anger and inability of the partner.	Woman. 'I perceive that my husband has issues with setting boundaries on his family. It's the reason why we argue, he gives everything to them and I don't feel like this is my home'.
Child dependency of the partner.	Woman. 'I perceive that my partner's family abuse him. They do not let him grow and they have him living inside a bubble. I would like him to have more confidence'.
Anger at the husband's link to the family.	Woman. 'I have been thinking about my anger in relation to how unfair his family treat him. He's in charge of their payments and needs. He tells me that since he's doesn't mind it, I shouldn't mind it either. His family don't support him and he does everything his family don't want to do'.
**Subcategory b. Causes of submission to the family**	
Indicators of child dependency.	Man. 'My main problem is unemployment, it's the reason why I can't form a home independent of my parents and overcome my fears and insecurities, which prevent me from growing. I feel suffocated by my father, he's extremely possessive'.

Table S2. *Risk factors of violence due to problems with the inner world projected on the other*

Elements found	Units of analysis and accounts
**Category 1: Overinterpretation of the other's behaviour**	
**Subcategory a. Paralysing fear**	
Internal fears that prevent regulating the other.	Woman. 'I'm afraid of his explosive temper, he's quite impulsive. He's never hit me or mistreated me but I fear him losing control'.
**Subcategory b. Painful manifestations when facing a real event and disagreement with the partner**	
Lack of communication and understanding from the other.	Woman. 'I feel assaulted and misunderstood by my husband regarding my illness (depression); he doesn't understand it, I feel it as an aggression'.

Despair at the other's behaviour.	Man. 'I worry about her illness, but she doesn't cooperate. She doesn't live life as it should be; I want her to be well. She's lives inside a bubble, it frustrates me'.
**Subcategory c. Impotence for not knowing how to behave**	
Impotence when facing a problem of the partner's.	Man. 'When we want to have sex, she pushes me off when I'm penetrating her. She pushes me away, I see her face and I don't feel any pleasure when I see her suffering'.
Attribution of health problems to couple problems.	Woman. 'I emotionally distanced myself from my partner some time ago. I begrudge him for I was diagnosed with diabetes and this caused me to lose my baby'.
The pain of a loss is transformed into anger.	Woman. 'Since I lost my babies when I was 3 months pregnant, I've been angry at everything because I begged God to get pregnant. I'm quite affected but my partner is acting normally, and I'm trying to take it out on him'.
**Subcategory d. Acting on assumptions**	
The other does not meet expectations.	Woman. 'He's never going to be the way I want him to be'.
The children become the repositories of the problems.	Woman. 'My husband wants to separate me from my family because I don't get along with his family. My little girl feels like her dad doesn't care and that he prefers his family'.
Intergenerational alliances.	Woman. 'My husband feels like I put ideas about his family in my daughter's head. My daughter no longer wants to see them. She's afraid of her dad because he's always mad at her due to this situation'.
Childish attitudes in the relationship with the partner.	Woman. 'If I need something, I ask for it through my daughter because if I ask him directly, he tells me I don't really need it and that I spend a lot of money'.
Overinterpretations.	Woman. 'Our biggest problem is misinterpretations. I have low tolerance to my husband's jokes because I never know if it's a joke or not'.
**Category 2: Relationship between the conflicts of their previous life and the conflicts with the partner**	
**Subcategory a. Internal voids**	
Form of escape-avoidance coping.	Woman. 'I'm afraid of criticism due to what my mother, my relatives and old schoolmates used to tell me, and now I fear the same from my husband and daughter. I have had to stay quiet and to swallow my problem. I feel like my life is quite lonely'.
Inability to defend oneself.	Woman. 'I've been a victim of the world's injustices. When I was nine, my younger cousin had an accident and passed away. I feel guilty and attacked by my family'.
**Subcategory b. Unresolved inner conflicts**	
Impotence when facing the attitudes of the other	Man. 'I felt like my wife is too close-minded. I don't like dancing but I got into it to share something with her. She's always living in fear'.
**Subcategory c. Inner dissatisfaction projected on the couple**	
Impotence when facing the wife's behaviour.	Man. 'I don't understand why my wife gets angry at the lack of control and order. She's stressed about things she shouldn't be stressed about. She does not accept any criticism and she doesn't tolerate to be told she can do something better'.
Impotence when facing the other's dissatisfaction.	Man. 'I sense that my wife is dissatisfied with our relationship. Nothing I ever do seems to be okay for her, I never bring her any happiness'.
Useless effort.	Man. 'I do my best to do things right but my wife criticises me at the slightest mistake. She attacks me and blames me'.
**Subcategory d. Problems projected on the children**	
Lack of regulation.	Man. 'I feel like my wife sees non-existent diseases in my daughter, however, she's at risk of getting diabetes and my wife doesn't watch what she eats. It bothers me because my wife is diabetic herself but I see no progress. I worry about my daughter becoming obese and envious when she's older'.

Table S3. *Risk factors of violence due to external reasons (Infidelity, infertility and perinatal losses)*

Elements found	Units of analysis and accounts
**Category 1: Repeated behaviours following the other's infidelity**	
**Subcategory a. Uncertainty**	
Inability to get out of the situation.	Woman. 'I checked his social media and found conversations that hurt me. I suspect he's cheating on me. I decided to separate but I can't do it and my husband keeps acting the same'.
Passivity Inability to make decisions.	Woman. 'I'm upset because of his infidelities. He doesn't pay for any home expenses. I told him to leave me alone and to go if he's not happy with me. He told me he doesn't know if he loves me'.
Psychological violence	Woman. 'We once talked on the phone and he didn't hang up properly and I heard him with someone else and he told her, 'Let's eat and then we'll be alone just the two of us''.
**Category 2: Identifying how they react to infidelity**	
**Subcategory a. Contradictions**	
Recurring ideas when facing a suspicion of infidelity.	Woman. 'I check his phone because I've heard he's cheating on me but he tells me I have no reason to check his things'.

Atrocities	Woman. 'I found one of my daughter's classmates with my husband. She's just a girl'.
Anger deposited on the victim.	Woman. 'All the fury my daughter feels towards her father after we broke up due to his infidelity is thrown at me. She blames me for pushing her father away'.
**Category 3: Risk factors triggered by infertility problems**	
**Subcategory a. Losing control over the other generates anguish**	
This promotes mistrust and control over the other.	Woman. 'I've been sad because my attempts at pregnancy haven't gone well. I can't be strong and my body doesn't respond to treatment. I don't see the results I wish for and I control everything all the time because I don't want my husband to leave me'.
**Subcategory b. External problems that occur due to infertility**	
Pain from infertility and abuse.	Woman. 'What I felt is over. Now I don't even want to see the light because I can't have children. My mother-in-law believes I'm the one with the problem and all responsibility has been thrown at me'.
The social surroundings worsen the pain.	Man. 'My family thinks my wife is the problem but I don't. I defend her from them and we don't feel support from my parents'.
Exhaustion caused by facing infertility.	Man. 'The chance of having a child seems out of reach now. We feel defeated and we're emotionally exhausted. We don't even have sex anymore'.
The sexual life is annulled.	Woman. 'There is no more sexual life between us. My husband no longer insists on it and we're both tired'.
Envy before what is considered unfair.	Woman. 'My husband's niece is pregnant. She's sixteen years old. I can't stand her and I can't tolerate her. I feel angry and resentful at her because she was able to get pregnant'.
**Category 4. Facing perinatal losses**	
**Subcategory a. Devastation caused by the losses**	
Fragility when facing a painful event.	Woman. 'I can't seem to get pregnant again. I've taken a lot of care of myself because I've had three miscarriages. The third one was a twin pregnancy. I never thought I'd suffer so much'.
Stages of grief	Man. 'We went on a family holiday and on our way there, I remembered my daughter who passed away. I thought, 'The baby could have been coming with us'. It hurts me like on the very first day'.
**Subcategory b. Impact on the couple relationship**	
Pain caused by the loss.	Man. 'I can't seem to accept the pain for the loss of my baby. I blame my wife for not taking care of herself during the pregnancy, she's diabetic'.
The pain of a loss is transformed into anger.	Woman. 'Since I lost my babies when I was 3 months pregnant, I've been angry at everything because I begged God for a baby. I'm quite affected but my husband is acting normally'.
The expression of pain as aggression.	Woman. 'I'm hurt by the loss of my baby. I can't express it and he never asks me about it. I felt a lot of loneliness. He's more interested in messing around than in me or his son'.

Table S4. *Risk factors that trigger intimate partner violence due to interaction*

Elements found	Units of analysis and accounts
**Category 1: Violence within the family of origin influences intimate partner violence**	
**Subcategory a. Intergenerational violence**	
Models of abuse during childhood repeated with the current family.	Woman. 'The problems I have with my family remind me of when my brother mistreated me. My mum didn't say anything. I come from a small town and we were taught to respect the eldest sibling. I see my husband acts the same way my mother used to and my son is like my brother because he mistreats my daughter'.
Repeated familiar models.	Man. 'I'm quite jealous but lately I've tried not to be. I used to be like my father and he used to be like my grandfather, they both were extremely possessive and aggressive. I want to break the cycle'.
Patterns of abuse repeated in adulthood.	Woman. 'During our first year together, I got angry and exploded on my husband, I even got to beat him. I once punched him. He only verbally assaulted me. I suffered a lot of violence during my childhood'.
Normalised family violence during childhood	Woman. 'My dad beat both me and my mother. My mother begged my grandmother to help her but my grandmother said it was her punishment. Nobody ever helped her and my dad threatened to kill me when I defended my mother'.
Mistreated grandmother, mother and daughter.	Woman. 'When my husband threw me out of our house, the memories flooded my mind because my grandmother went through the same thing and it feels like history repeats itself'.
**Subcategory b. Maternal rejection**	
Abusive mother due to dissatisfaction	Man. 'My mother didn't want me. I don't understand why she brought me to the world if she didn't want to have me. I distanced myself from her and she didn't love my father'.
Consequences of the domestic violence.	Woman. 'My mother abandoned us when I was eight because my father beat her. I don't have any nice memories of them'.
Emotional deficiencies during childhood and resentment.	Woman. 'I resent my mother because she was never affectionate towards us. She struggled to tells us she loved us and she didn't give us any affection'.

Rejecting the mother for being a woman; defencelessness.	Woman. 'I think of my mother as a child. I resent and feel angry at her. She used to mock and punish me. I hated being a woman'.
The conflict with the mother is repeated with the daughter.	Woman. 'The relationship with my mother is ambiguous: it's both warm and rude. I've repeated this with my daughter. She mistreats me but if I stay quiet it is worse'.
**Category 2: Escalation of violence.**	
**Subcategory a. Symmetrical violence**	
Polarized relationships.	Woman. 'My relationship is extreme. Sometimes we love each other and sometimes we hate each other. Our arguments grow increasingly nasty. I've punched him twice'.
**Subcategory b. Asymmetrical violence (one subjected to the other)**	
Causes of entrapment in an abusive relationship.	Woman. 'I feel anxious at my situation because I can't find a way to separate from my husband. The abuse continues and I face him about it, but I can't break away. I'm anxious at the thought of becoming independent. My husband insults me'.
Impotence when facing abuse.	Woman. 'I feel like a prisoner in my own house. Sometimes I don't even want to go back. Therapy makes me feel alive. I'm trying to change to feel better but my husband tells me that if I submit myself to him, we'll be fine. I want to be free'.
One imposing over the other.	Woman. 'I ask my husband for respect but he tells me that he's the number one priority in the family, followed by our children and I'm the last one'.
Fear, insecurity, feelings of devaluation.	Woman. 'From the beginning of our marriage, he was controlling. He punched me. I feel dumb and I'm not very cunning. I'm fearful and insecure'.
Inability to modify their life.	Woman. 'I keep on telling him I want to go out but he doesn't give me any money. I want to work but he says no. He hurts me. I've been with him for 46 years and he's never respected or valued me'.
Exclusion in collusion with the children.	Man. 'I'm a waste of space for my family. If I scold or talk to my children, my wife immediately belittles me before them. I prefer to stay quiet'.
Submission when facing intimate partner violence.	Woman. 'He tells me he no longer likes me sexually and that I disgust him if I don't submit myself to him the way he wants to. He calls me crazy when I complain to him about his lover'.
Domineering attitudes from an authoritarian woman.	Man. 'I give everything I have to my wife and children. She administers the money we both earn. Nevertheless, if I take some for an emergency, my wife complains, abuses me and treats me like her servant'.
Resentments poured on the partner.	Man. 'I feel like whatever I do, I can't seem to make my wife happy. She criticises everything about me and she's bothered by everything. I asked for a water bottle and she refused'.
**Subcategory c. Identifying elements that are triggered when on tries to be or becomes autonomous**	
Abuse when autonomy is achieved.	Woman. 'I had an argument with my husband but I no longer give in, I defend myself. I love him but I won't stand for this. He doesn't like me going to my yoga lessons. He's indifferent towards me and doesn't talk to me'.
Authoritarianism and absolute control.	Woman. 'I had a row with my husband because I began taking swimming lessons. He told me that if I was under his care, I must accept his rules. I shouted at him and called him misogynist, and he hit me'.
Harassment, control.	Woman. 'I set boundaries on him and ask him to respect me. He believes that I cheat on him whenever I go out. He tells me what to wear and I feel 'tied' to him. He's violent and believes himself to be in charge. He restricts my money and harasses me. I'm not free'.
Control and isolation.	Woman. 'My husband doesn't let me come to therapy. He's aggressive and threatens me with hurting my mother. He doesn't let me visit her and he wants to control me'.
Increase of violence due to changes in the victim.	Woman. 'My husband tells me that I'm no longer submissive or feminine and that I'm not coming back to therapy. I tell him I'll come as long as I have to. He wants to impose his views on me and he tells me to respect him because I'm acting like a man. I want to make my own decisions'.
**Category 3. Tolerating abuse**	
**Subcategory a. Repercussions in children**	
Passivity when facing the other's aggressions towards the children.	Woman. 'The best thing to do is to help one's children with love. My husband threw my daughter out and she hasn't come back home. I feel guilty, I couldn't stop her'.
Limitless efforts to please to other.	Woman. 'I organised all the family outings when my children were young so he would only worry about petrol. I told him I didn't want to fight because we were going out with the children. He put conditions on my money'.
Inability to set boundaries.	Woman. 'My daughter resented all the violence in our relationship when she was a child. She suffered anorexia and bulimia. She also tried to commit suicide, she cut her wrists. I didn't set any boundaries'.
**Subcategory b. Factors that prevent the separation of one member of the couple despite wishing for it**	
Factors that prevent decision-making.	Woman. 'I have reflected on the reasons that keep me tied to my husband in spite of our troublesome relationship. I'm afraid of that people will say and I fear how complicated it would be to economically support myself. I get very anxious'.
Submission as a high-risk characteristic in the couple.	Woman. 'I don't feel at ease with my husband. He's unfaithful and abuses me, and I'm afraid of him assaulting me and my family. I feel like I can't go on'.

**Category 4: Abuse that goes beyond the couple relationship**	
**Subcategory a. Self-abuse and abuse towards the family**	
Self-abuse.	Woman. 'I'm aware of the things that hurt me but I can't control it. I must have something at all costs. I've put on around 45 pounds in 6 months and I'm diabetic'.
Devaluation, self-abuse and self-destructive fantasies.	Woman. 'My sister passed away because of her diabetes. I thought that maybe I should have died instead of her'.
Lack of self-regulation.	Man. 'I felt really bothered by my wife losing her mobile. I tried to control myself because I don't want to be like my father'.
**Subcategory b. Physical separation doesn't always change the bond**	
The separation not always changes the couple dynamics.	Woman. 'I've ruined the coexistence with my family particularly when I prevent my husband from leaving. I hook onto him in spite of us being separated. I keep having a conflictive relationship with him'.
Repetitive behaviours in spite of the separation.	Woman. 'I feel like I can't stop my husband. He's always been like that and we went back to the dynamics we had before separating. He asked me to live together again but I don't want to go back to him'.
**Subcategory c. Normalising violence**	
Normalisation of violence.	Woman. 'My relationship lacks several elements: trust, communication and respect. I've lived like this for so long that it feels normal by now'.
Starting to uncover attitudes.	Woman. 'I can now see the truths I was avoiding. I'm realising that a partner is a complement and that he wanted to control everything'.
